# Correction: Silva et al. Precision Technologies to Address Dairy Cattle Welfare: Focus on Lameness, Mastitis and Body Condition. *Animals* 2021, *11*, 2253

**DOI:** 10.3390/ani12060683

**Published:** 2022-03-09

**Authors:** Severiano R. Silva, José P. Araujo, Cristina Guedes, Flávio Silva, Mariana Almeida, Joaquim L. Cerqueira

**Affiliations:** 1 Veterinary and Animal Research Centre (CECAV), Associate Laboratory of Animal and Veterinary Sciences (AL4AnimalS), University of Trás-os-Montes e Alto Douro, Quinta de Prados, 5000-801 Vila Real, Portugal; ssilva@utad.pt (S.R.S.); cguedes@utad.pt (C.G.); fsilva@uevora.pt (F.S.); mdantas@utad.pt (M.A.); 2 Escola Superior Agrária do Instituto Politécnico de Viana do Castelo, Rua D. Mendo Afonso, 147, Refóios do Lima, 4990-706 Ponte de Lima, Portugal; pedropi@esa.ipvc.pt; 3 Mountain Research Centre (CIMO), Instituto Politécnico de Viana do Castelo, Rua D. Mendo Afonso, 147, Refóios do Lima, 4990-706 Ponte de Lima, Portugal

The authors (Silva, S.R., et al.) unintentionally omitted to cite the article by O’Leary et al. “O’Leary, N.W.; Byrne, D.T.; O’Connor, A.; Shalloo, L. Invited review: Cattle lameness detection with accelerometers. *J. Dairy Sci.* **2020**, *103*, 3895–3911. doi: 10.3168/jds.2019-17123” and unintentionally included two passages (9 sentences) without quotation marks from O’Leary et al. 2020 in the original. Therefore, these two passages were inadvertently presented as the work of the authors. Authors wish to apologize to the authors of the uncited work and to make the following corrections to [1]: In the original article, Page 5, Section 2.1.3, “Activity-Based Techniques”, the sentences “Westin et al. [75] concluded that only a small proportion of variation in lying time could be explained by lameness. In aggregate, measures of lying time are not reliable indicators of lameness, partly because lying time is influenced by many factors other than lameness. For these reasons, further research focusing on measures of lying time alone to support automated lameness detection is unlikely to be successful” and “The first accelerometer-based automated lameness detection system was marketed by IceRobotics (Edinburgh, UK) in 2017 [77], and locomotion scoring also was marketed. The system is based on a single low resolution accelerometer per cow. The system presents users with the probability that a cow is lame using a traffic light system. Cows that are likely to be non-lame are green, those that may be lame are yellow, and those likely to be lame are red [77]. This approach is different from those seen in the literature, but may be an appropriate solution for communicating information with less than perfect accuracy to farmers” should read in the corrected version:

“In a recent comprehensive work on detecting lameness in cattle, O’Leary et al. [75] support results from another report [76] that show the length of lying time is not a reliable indicator because it only explained a small proportion of the variation of lameness in dairy cows as lying time is influenced by many other factors. For these reasons, further research to support automatic lameness detection needs to focus on aspects other than lying time measures to succeed [75]” and

“In recent years, the development of accelerometer-based automated lameness detection systems has continuously evolved [75]. The first system was marketed in October 2018 by IceRobotics (Edinburgh, UK) [78]. In this system, each cow is equipped with a single low-resolution accelerometer. The system presents users with simple information similar to the traffic light system with the colors green, yellow and red if the cows are identified as likely to be non-lame, maybe lame, or those likely to be lame, respectively [78]. This approach can be very suitable for straightforward communication to farmers [75]”.

In addition, the authors wish to make the following correction to this paper [1]:

**Section 2.1.** “Lameness is mainly detected at an advanced stage and thus requires immediate and often costly treatment. Once an animal becomes lame, it can take several weeks to recover, thus representing a high cost to dairy farmers in terms of time, financial expenditure for veterinary calls, medication, and treatment [29]. Time constraints for dairy farmers are an essential factor contributing to the under-detection of lameness, resulting in delayed or missed treatment of lame cows. Hence, a need exists for flexible and affordable cow-based sensor systems capable of recording behaviors such as time spent feeding, which may be affected by the onset of lameness [30].” needs to be replaced by “Lameness is usually detected when the disease is already at an advanced stage and requires immediate and often expensive treatment. An animal in these circumstances can take several weeks to recover, representing a high cost for dairy farmers in terms of time, financial expenses for calls to the veterinarian, medication and treatment [29]. Time limitations of the dairy producers is a factor that contributes to the under-detection of lameness problems. Therefore, using flexible and affordable sensor-based systems is a need for recording the cows’ behavior and thus identify the onset of lameness [30]”.

**Section 2.1.2.** “Remote sensing solutions such as 2D or 3D video cameras have excellent potential as lameness monitoring systems. However, there are challenges when developing algorithms for such devices, as one algorithm has to work for multiple animals even though individual cows have their specific way of walking and their lameness is expressed in a particular way. To meet this challenge, real-time lameness detection systems must account for the normal healthy behavior of the cow so that abnormalities can then be picked up quickly. Such an approach requires maximizing the usage of historical and real-time data. Individualized monitoring systems using animal-level historical data have achieved better detection accuracy than population-based monitoring systems. Back posture values, automatically extracted from top view 3D images of the cows’ back, are used to measure the degree of lameness [64]. One back posture value can indicate lameness for one cow but soundness in gait for another. This individual cow variation has already been pointed out in previous research and confirms that back posture values should be analyzed and interpreted at an individual cow level and that a healthy reference should be calculated for each cow separately [65].” needs to be replaced by “Solutions with 2D or 3D video cameras have the potential to be applied in lameness monitoring systems. Considering the character individual of normal and lame walking of the cows, however, challenges arise with the development of algorithms that must work broadly for all cows. Real-time lameness detection systems must consider normal and healthy behavior to detect abnormalities immediately to overcome this challenge. Typically in the 2D and 3D image system, the back posture is examined to measure the degree of lameness, and values are automatically extracted from a top view of the cows [64]. However, as mentioned previously, the back posture shows individual cow variation, indicating lameness for one cow but normal gait for another. Thus, cow posture values must be analyzed individually and compared with what is considered normal for each cow separately. The analysis of historical and real-time data from a given animal allows tuning a model to a healthy reference behavior in the case of lameness monitoring [65]”.

**Section 2.1.3.** “Beer et al. [76] reported relatively accurate lameness detection based on an accelerometer-based estimation of speed, stride length, and duration, and reported that lame cows walked more slowly and with shorter stride lengths than non-lame cows, using data from only one 10-Hz accelerometer per cow. A sensitivity of 90.2% and a specificity of 91.7% were reported using both gait and behavior measures. Measuring acceleration at the level of the metatarsus, using two accelerometers with a high sampling rate (400 Hz) attached to both hind limbs, is a promising tool for exploring the acceleration of the lateral claw indirectly, and for accurately describing the different gait cycle variables [44].” needs to be replaced by “In this sense, other authors [77] developed a model for automatic lameness detection using data from an accelerometer-based approach applied to multiparous Holstein lame (n = 41) and non-lame (n = 12) cows. This work showed that lame cows show shorter strides and a slower walking speed than non-lame cows and that the best model to detect cows being lame considers the number of standing bouts and walking speed with a sensitivity of 90.2% and specificity of 91.7%. Also, measuring acceleration at the metatarsal level with accelerometers in each of the hind limbs proved to be a promising tool to describe the different variables of the gait cycle accurately [44]”.

**Section 2.1.4.** “Change in an animal’s behavior is one of the most important criteria in assessing animal welfare and health. For example, pain associated with claw or limb disorders causes alterations in gait characteristics and a decreased daily activity level [76]. Additional placement of different sensor types in the same body location (e.g., rumination audio sensor, magnetometer) or an additional accelerometer in an alternative body location (e.g., leg-mounted) would likely be needed to accurately classify the three main behaviors of interest in dairy cows (lying, standing, and feeding) [30,83]. Analysis of the classified behavior highlights differences in feeding activity, with feeding duration being significantly lower for lame cows than non-lame cows. The results highlighted how automated collection of behavioral data via a combined position and activity sensor could potentially form part of an on-farm health and welfare monitoring tool [30].” needs to be replaced by “Evaluating change in an animal’s behavior is one of the most used criteria to assess its health and welfare. A good example is given by the pain linked with diseases of the claws or limbs of dairy cows, which produce changes in movement pattern and a decrease in daily activity [77]. Using diverse sensor types in different body locations (e.g., neck or leg-mounted) would be required to correctly classify lying, standing and feeding, which are key behaviors in dairy cows [30,84]. For example, Barker et al. [30], who used automated behavioral data collection through a combined position and activity sensor, observed a shorter feeding duration for lame cows than non-lame cows. This result shows that behavior analysis can be a tool for monitoring the health and welfare of cows [30]”.

**Section 2.2.** “Mastitis is one of the most common diseases in dairy cows and causes suffering in affected animals, which has well-recognized detrimental effects on welfare and dairy farm profitability [85,86]. Therefore, since the beginning of modern dairy farming, producers have sought effective methods to minimize mastitis in their herds. The development of a control program incorporating post-milking teat dipping, hygienic milking procedures, and strategic use of antibiotic therapy in dry-off resulted in widespread control of contagious pathogens. However, as mastitis pathogens have evolved, researchers have sought to control antimicrobial usage to maintain animal wellbeing, while minimizing unnecessary usage.” needs to be replaced by “One of the most relevant diseases in dairy cows is mastitis, a cause of suffering in infected animals, with worldwide recognized harmful effects on the welfare and profitability of dairy farms [86,87]. Thus, producers have been concerned with implementing effective methods to control mastitis in their herds since the first mechanized milking systems appeared. The development and implementation of control programs that integrate pre and post-milking teat immersion, correct milking procedures and restricted use of antibiotics in drying only in infected cows have resulted in a significant decrease in contagious pathogens. However, as mastitis pathogens emerged, researchers sought to restrict the use of antimicrobials while preserving animal welfare and respecting universal guidelines for unnecessary use”.

“Efficient mastitis detection provides an opportunity to implement early and adequate treatment protocols and avoid excessive use of antibiotics, maintaining good animal health and welfare by reducing pain and discomfort, enhancing recovery rate, and improving economic returns to farmers [88,89].” needs to be replaced by “Reliable detection of mastitis through automated methods represents an excellent opportunity to carry out early treatment programs and avoid overuse of antibiotics, preserving the health and welfare of cows, avoiding discomfort and pain, improving the recovery rate and the economic sustainability of farms [89,90]”.

**Section 2.2.1.** “Management of udder health is essential for maintaining efficient and sustainable dairy production. Somatic cell count (SCC) is a widely used indicator of udder health status in dairy cows and is used at the quarter, cow, and bulk-tank levels. In automatic milking systems (AMS), fully automated online analysis equipment is available for on-farm analysis of SCC at every milking [92]. In addition, from online cell counter results, an array of additional cow level and quarter-level factors considered important for udder health is recorded in these systems [93].” needs to be replaced by “Health management is essential for maintaining efficient and sustainable dairy production. Somatic cell count (SCC) is the most used indicator to assess udder health status in dairy cows, being used at a quarter, cow and bulk tank levels. In automatic milking systems (AMS), fully automated online analysis equipment is available to analyze SCC at the farm at each milking [93]. Moreover, from the results of the online SCC, a number of additional cows and quarter level factors important for udder health are recorded in these systems [94]”.

“This represents a substantial increase in the amount of data, e.g., for udder health management, and which may also serve as phenotypes for breeding programs. In addition to frequent measurements of SCC, a whole array of additional cow-level and quarter-level factors considered of importance for udder health are recorded in the AMS at every milking [95].” needs to be replaced by “This represents a considerable increase in the amount of data, for example, for udder health management, which can also serve as phenotypes for reproductive programs. In addition to the frequent measures of SCC, a number of additional cow level and quarterly factors considered of importance for udder health are recorded in the AMS in each milking [96]”.

**Section 2.2.2.** “Currently, an increasing number of dairy farmers worldwide choose AMS, which allow farmers to maximize milking frequency, potentially milk production per cow, and minimize labor costs [98]. In AMS, the sensors that measure EC are the in-line sensors most commonly used to detect mastitis. These sensors can continuously measure the concentration of ions in milk during the milk harvesting process, albeit with variable results [99]. Foremilk sampled before milk ejection was more sensitive for detection of mastitis than foremilk harvested after milk ejection; due to udder preparation, including teat cleaning in AMS systems. In addition, both LDH activity and milk protein contents were higher in quarters with Gram-negative coliform mastitis than in quarters with mastitis caused by Gram-positive bacteria. These results suggest that, in the future, sensors could be modified to monitor milk removed before teat cleaning, to improve the ability of AMS to detect mastitis [99].” needs to be replaced by “In recent years, there has been an increasing choice of AMS worldwide. This type of equipment allows producers to increase milking frequency, milk production per cow and reduce labor costs [99]. The AMS is equipped with in-line sensors that measure EC to detect mastitis. These sensors make a continuous assessment of the concentration of milk ions during the milk collection process. However, the results are variable, with the first milk collected before milk ejection being more sensitive to detecting mastitis than the first harvested milk, which is explained by udder preparation and teat cleaning in AMS systems [100]. For this reason, it is pointed out that in the future, to improve the ability of AMS to detect mastitis, sensors should monitor the milk before teat cleaning [100]”.

**Section 2.3.** “BCS can be done using only visual indicators or a combination of visual and tactile estimation of key bone structures for fat cover. The key areas or body parts for BCS assessment are the backbone, pins, tail head, long ribs, short ribs, hips, and rump [105]. Over the years, different scoring scales have been developed around the world. For example, in the USA, a five-point scale system, proposed by Windman et al. [110], was commonly used, where BCS value varies from 1 to 5.” needs to be replaced by “BCS assessment can be performed by visual assessment or by a combination of visual indicators with palpation of bone structures and the degree of subcutaneous fat. The key areas for BCS assessment are the backbone, pins, tail head, long ribs, short ribs, hips, and rump [106]. Over the years, different scoring scales have been developed around the world. For example, a five-point scale system was commonly used in the USA, proposed by Windman et al. [111]”.

The authors apologize for any inconvenience caused and state that the scientific conclusions are unaffected. The original article has been updated.

**New Reference**

[75] O’Leary, N.W.; Byrne, D.T.; O’Connor, A.; Shalloo, L. Invited review: Cattle lameness detection with accelerometers. *J. Dairy Sci.* **2020**, *103*, 3895–3911. https://doi.org/10.3168/jds.2019-17123.
